# Gamma Delta TCR and the WC1 Co-Receptor Interactions in Response to *Leptospira* Using Imaging Flow Cytometry and STORM

**DOI:** 10.3389/fimmu.2021.712123

**Published:** 2021-07-28

**Authors:** Alexandria Gillespie, Maria Gracia Gervasi, Thillainayagam Sathiyaseelan, Timothy Connelley, Janice C. Telfer, Cynthia L. Baldwin

**Affiliations:** ^1^Department of Veterinary & Animal Sciences, University of Massachusetts, Amherst, MA, United States; ^2^Roslin Institute, University of Edinburgh, Edinburgh, Scotland; ^3^Program in Molecular & Cellular Biology, University of Massachusetts, Amherst, MA, United States

**Keywords:** gamma delta T cells, gamma delta TCR, WC1, STORM, *Leptospira*

## Abstract

The WC1 cell surface family of molecules function as hybrid gamma delta (γδ) TCR co-receptors, augmenting cellular responses when cross-linked with the TCR, and as pattern recognition receptors, binding pathogens. It is known that following activation, key tyrosines are phosphorylated in the intracytoplasmic domains of WC1 molecules and that the cells fail to respond when WC1 is knocked down or, as shown here, when physically separated from the TCR. Based on these results we hypothesized that the colocalization of WC1 and TCR will occur following cellular activation thereby allowing signaling to ensue. We evaluated the spatio-temporal dynamics of their interaction using imaging flow cytometry and stochastic optical reconstruction microscopy. We found that in quiescent γδ T cells both WC1 and TCR existed in separate and spatially stable protein domains (protein islands) but after activation using *Leptospira*, our model system, that they concatenated. The association between WC1 and TCR was close enough for fluorescence resonance energy transfer. Prior to concatenating with the WC1 co-receptor, γδ T cells had clustering of TCR-CD3 complexes and exclusion of CD45. γδ T cells may individually express more than one variant of the WC1 family of molecules and we found that individual WC1 variants are clustered in separate protein islands in quiescent cells. However, the islands containing different variants merged following cell activation and before merging with the TCR islands. While WC1 was previously shown to bind *Leptospira* in solution, here we showed that *Leptospira* bound WC1 proteins on the surface of γδ T cells and that this could be blocked by anti-WC1 antibodies. In conclusion, γδ TCR, WC1 and *Leptospira* interact directly on the γδ T cell surface, further supporting the role of WC1 in γδ T cell pathogen recognition and cellular activation.

## Introduction

γδ T cells in many mammals exclusively express cell surface molecules known as T19 or WC1 (1–3) that are part of the Group B scavenger receptor cysteine rich (SRCR) superfamily. They are coded for by multigenic arrays that are largely conserved throughout evolution (4–6). While neither humans nor mice have WC1 they do express the closely related SRCR molecules CD163 and CD163c-α on their γδ T cells (7, 8) with many SRCR domains being homologous between WC1 and CD163 family members (9). In particular, mice express the variants SCART1 and SCART2, known to be involved in functional subset differentiation of murine γδ T cells (8, 10). Also, scavenger receptors in general play important roles in immune responses by binding pathogens. This includes immune system cells’ SRCR molecules CD5 that binds fungi, and CD6 and CD163 that bind bacteria (11–13). Similarly, we have shown that specific SRCR domains of WC1 bind *Leptospira* and that single point mutations can disrupt the binding (14). Thus, Group B SRCR family members act as pathogen recognition receptors (PRR).

We have shown that WC1 also acts as a T cell receptor (TCR) co-receptor on γδ T cells somewhat akin to CD4 and CD8. This is based on the observation that when co-crosslinked with the TCR complex it augments activation, while when WC1 is ligated alone there is no activating effect and can cause G0-arrest (15–17). This co-receptor function is mediated by signal transduction as a result of phosphorylation of specific tyrosines in the WC1 intracytoplasmic domains that can be disrupted by point mutations (16, 18, 19). Importantly, shRNA-mediated knockdown of WC1 significantly inhibits the ability of bovine γδ T cells to respond to *Leptospira* (20). In addition, monoclonal antibodies (mAb) reactive with the TCR can block WC1^+^ γδ T cell responses (21–23). Therefore, both TCR and WC1 are necessary for responsiveness.

The bovine γδ T cells can be divided into subpopulations WC1.1^+^ and WC1.2^+^ as a result of variegated gene expression of WC1 molecules. These can be distinguished by monoclonal antibody (mAb) reactivity to the WC1 variants (24) and their reactivity to pathogens. Cells within the WC1.1^+^ subpopulation proliferate and produce interferon (IFN)γ in response to the zoonotic pathogen *Leptospira*, while most cells in the WC1.2 subpopulation do not respond (25). Moreover, the WC1 molecules expressed by the leptospira-responsive cells physically bind *Leptospira*, while the WC1.2 variants do not (14). However, WC1.2^+^ cells respond to other pathogens including *Mycobacteria* and *Anaplasma* (21, 22). Thus, it is hypothesized the tailored responses are at least partially a result of the ability of particular WC1 variants expressed by the cells to interact with the pathogen.

Based on the results showing the involvement of WC1 both in pathogen recognition and TCR-dependent signal transduction, we hypothesized that WC1 and TCR will physically interact by co-localization when the cells are ligated by antigen. To examine this hypothesis, we used imaging flow cytometry (AMNIS) and stochastic optical reconstruction microscopy (STORM), evaluating the localization of WC1 variants and the γδ TCR on quiescent cells and cells activated with the bacterial pathogen *Leptospira* in recall responses *in vitro*. We found γδ TCR and WC1 molecules formed clusters or protein islands that result in fluorescence resonance energy transfer (FRET) following activation but not on quiescent cells. These clusters excluded CD45. We also showed specific binding of *Leptospira* to the WC1 co-receptors expressed on the cell membrane further supporting the role of WC1 in γδ T cell pathogen recognition and cellular activation.

## Materials And Methods

### Animals and Cells

Blood was collected into heparin from the jugular vein of cattle (n=2) in accordance with the protocol approved by the IACUC of the University of Massachusetts Amherst. Both animals were vaccinated against *Leptospira* serovar hardjo with the commercial whole cell inactivated vaccine *Spirovac* (Pfizer). PBMC were isolated from blood by centrifugation over ficoll-hypaque and suspended at 2.5x10^6^ cells/ml in complete RPMI (RPMI-1640 with 10% heat-inactivated fetal bovine serum (FBS, Hyclone), 5x10^-5^M 2-mercaptoethanol, 200 mM L-glutamine (Sigma), and 10mg/ml gentamycin (Invitrogen)). Where indicated PBMC were cultured with 0.08 ug/ml of sonicated *Leptospira* or whole *Leptospira* for 1 hr to 7 days as indicated.

### Immunofluorescence

Cells were stained by indirect immunofluorescence with primary mAb GB21A (anti-TCRδ), CC15 (anti-pan-WC1), CACTB32A (anti-WC1.2), BAG25A (anti-WC1.1), CACT21A (anti-WC1-8, marking WC1.3^+^ cells), MM1A (anti-CD3), GC42A (anti-CD45), IL-A29 (anti-pan-WC1), ILA-12 (anti-CD4), and ILA-51 (anti-CD8) or with cholera toxin subunit B (to mark lipid rafts). Appropriate secondary goat anti-mouse isotype-specific antibodies were used conjugated with one of the following fluorophores: Alexafluor647 (AF647), Alexafluor488 (AF488), or pycoerythrin (PE) as indicated. All commercially available isotype-specific secondary antibodies (Southern Biotech) have been cross-absorbed against all other mouse Ig isotypes and tested for specificity in our hands. Product numbers of secondary antibodies conjugated with PE are 1080-09, 1070-09, 1020-09; with AF647 are 1080-31, 1070-31, 1090-31; and with AF488 are 1091-30, 1070-30. Controls included secondary antibodies alone with cells. To assess cell proliferation, PBMC were loaded with efluor670 (Invitrogen) in accordance with the manufacturer’s protocol prior to culture. For AMNIS experiments, all primary and secondary antibodies were titrated to avoid background fluorescence.

### Imaging Flow Cytometry and FRET

Cells were fixed with 4% paraformaldehyde following immunofluorescence staining before being analyzed by imaging flow cytometry using AMNIS Imagestream Mark II. Results were analyzed using the AMNIS IDEAS software. FRET was assessed using the donor and acceptor fluorophore combination of PE (Blue laser 488) and AF647 (Red laser 642), respectively. Compensation matrices for FRET experiments were obtained with all lasers on (i.e., Red 642, Blue 488, Violet 405, SSC 785) using a sample with no fluorescence in the acceptor channel as well as samples with fluorescence in the acceptor channel. Juxtaposition of cholera toxin B with cell surface proteins was assessed with the colocalization Wizard within the IDEAS software package. Aspect ratio displayed in figures is calculated by the AMNIS software as the ratio of the length of an individual cell’s major axis and minor axis.

### STORM

Following staining of cells by immunofluorescence, cells were placed onto glass coverslips coated with poly-L-lysine for 1-2 hours before or after fixing with 4% paraformaldehyde followed by washing. Imaging buffer (690 µL Buffer B containing 50mM Tris at pH 8.0, 10mM NaCl, and 10% glucose) with 7 µL 2-mercaptoethanol and 7 µL GLOX solution [14 mg glucose oxidase, 50 µL catalase, and 200 µL Buffer A (10mM Tris, and 50mM NaCl)] was placed directly over adhered cells. Images were acquired in a Nikon N-STORM microscope using a Nikon PlanApo 100x NA 1.36 objective. To achieve super-resolution, a total of 20,000 images were collected in a sCMOS camera at a rate of 99 frames/sec. Exposure time was 10 millisec. Single molecule localization, reconstruction and cross-correlation for drift correction were performed using the FIJI image J ThunderSTORM plugin (26). The localization precision was 92 ± 22.4 nm for *ex vivo* cells and 90 ± 22.0 nm for cultured cells. Pearson’s coefficient of colocalization was used as recommended (27) to assess relative fluorescence from two channels and analyzed using the corr2 MATLAB function with 8-bit super-resolution reconstructions from ROIs of each cell from each channel to achieve a range of values from non-correlated (value = zero) up to perfectly correlated (value = one).

### Bacterial Binding

*Leptospira interrogans* serovar hardjo strains 1343 and 818 spirochetes were cultured in enriched Ellinghausen McCullough Johnson Harris medium (Sigma) at 30°C for 2 months, splitting cells to a concentration of 5x10^7^/ml every 2 wks. Bacterial concentration was determined by OD 600 (1 OD 600 = 8x10^8^ bacteria/ml). Spirochetes were fixed with 8% paraformaldehyde for 2 hrs and washed with PBS before biotinylation using the EZ-link sulfo-NHS-LC-biotin (Thermofisher) in accordance with the manufacturer’s protocol for biotinylating cell surface proteins (200 µL of 10 mM biotin per 2.5x10^7^ cells for 30 min). Unbound biotin was quenched with 100mM glycine solution in PBS. Biotinylated bacteria were then pre-stained with either Streptavidin-PE or Streptavidin-AF488 as indicated before use in binding experiments.

For evaluating binding of bacteria to lymphocytes, the labeled bacteria were incubated with bovine PBMC at a concentration of 2 to 2.5 x10^6^ bacteria per 5x10^6^ cells in a volume of 1 ml for 2-3 hr at 37°C with agitation every 30 min for flow cytometric analysis [FACS ARIA (BD)] to assess binding of bacteria to the cells. For flow cytometry sorting to enrich for WC1^+^/*Leptospira* ^+^ cells for STORM analysis the volume was reduced to 0.25 ml with the same number of bacteria and lymphocytes and PBMC were stained by indirect immunofluorescence with mAb CC15 (anti-pan-WC1) after incubation with the bacteria. The CC15^+^ PBMC with bound *Leptospira* were sorted to a purity of 59.9% (the low percentage is a result of bacteria being released from the lymphocytes during the sorting process). Assessing blocking of *Leptospira* binding by antibodies was performed by staining of lymphocytes with the indicated mAb and isotype specific secondary Ab conjugated with fluorochrome either before or after a 3 hr incubation with pre-stained bacteria with several washes between steps. Flow cytometry was then used to determine the percentage of mAb-stained cells binding fluorescent bacteria. The results were expressed as the ratio of cells binding bacteria with and without mAb blocking.

### Crosslinking WC1 and TCR

Bovine PBMC were stained with mAb GB21A (anti-TCRδ, IgG2b) and IL-A29 (anti-pan-WC1, IgG1) and secondary antibodies goat anti-mouse IgG, goat anti-mouse IgG2b, and/or goat anti-mouse IgG1 as indicated. They were cultured in 96-well plates with 1.25 x 10^5^ cells/well in a total volume of 200 µl. Cultures were established in triplicate for each condition and incubated for 4 days that included an overnight incubation with ^3^H thymidine (New England Nuclear) with 1µCi/well added on day 3 of culture. Cells were harvested with a cell harvester, incorporation of ^3^H thymidine determined by liquid scintillation and results expressed as counts per minutes (CPM).

### Statistical Analyses

For all FRET comparisons a 2-way ANOVA was performed followed by 1-way Student’s t-test for those showing differences. For co-localization Pearson’s correlation coefficient was employed and Student’s t-test of cells that were analyzed. For blocking of *Leptospira* binding to cells by antibodies, a Mann-Whitney rank sum test was used since the data were not normally distributed as a result of differences in bacterial binding efficiency among experiments. Significance is indicated as *p≤ 0.05 **p ≤ 0.01. ^3^H-thymidine results are shown as mean and standard error of the mean.

## Results

### WC1 and γδ TCR Physically Interact After Activation by *Leptospira*

**Figure 1:**
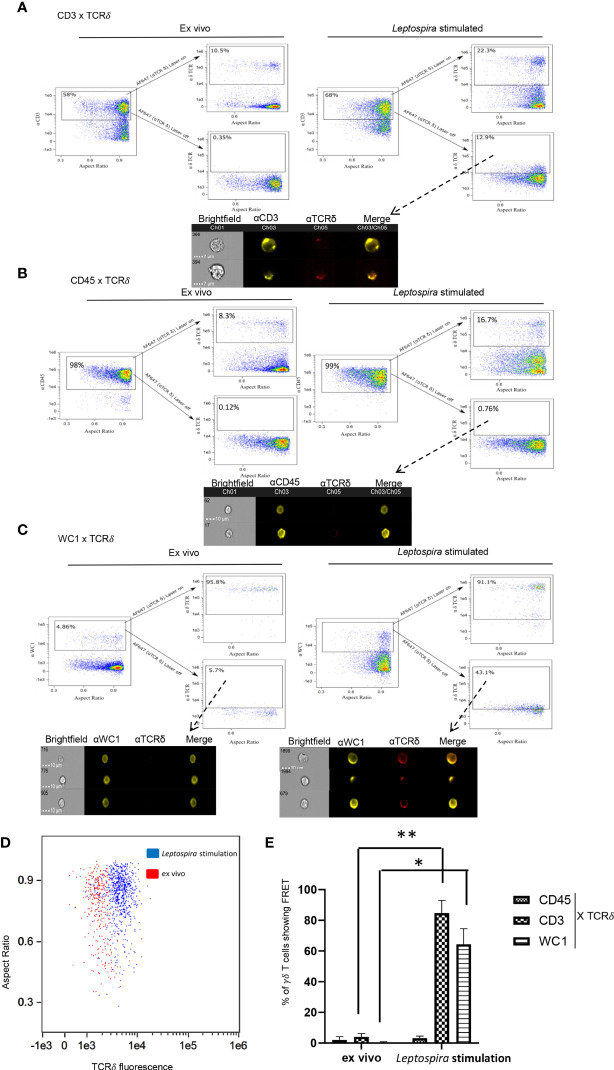
FRET between cell surface molecules. AMNIS imaging flow cytometry of bovine PBMC either quiescent or after culture with *Leptospira* antigen for 7 days. For **(A–C)** the top flow cytometry plots after the arrow indicate fluorescence with both lasers on, while the bottom panels show fluorescence with the red AF642- laser off to measure fluorescence-sensitized emission in that channel. Individual cell pictures from the indicated (from dashed arrow) gated population are also shown. Stained by indirect immunofluorescence with **(A)** anti-CD3 mAb with anti-IgG1-PE Ab (donor) and anti-TCRδ mAb with anti-IgG2b-AF647 Ab (acceptor), **(B)** anti-CD45 mAb with anti-IgG2a-PE Ab (donor) and anti-TCRδ mAb with anti-IgG2b-AF647 Ab (acceptor), or **(C)** anti-WC1 mAb CC15 with anti-IgG2a-PE Ab (donor) and anti-TCRδ mAb with anti-IgG2b-AF647 Ab (acceptor). Dot plots are representative of 3 independent experiments for **(A–C)**. **(D)** TCR acceptor fluorescence of WC1^+^ cells as a result of anti-WC1 (donor) mAb and anti-TCRδ mAb (acceptor) interaction on *ex vivo* resting cells (red dots) and *Leptospira* stimulated cells (blue dots). **(E)** The mean ± SD percentage of cells showing FRET relative to the maximal number possible from 3 independent experiments are shown. No significant FRET was found for CD45 interaction with TCR but it was for CD3-TCR and WC1-TCR interactions (*p ≤ 0.05 **p ≤ 0.01 by Student’s t-test: CD3 *p* = 0.005, WC1 *p* = 0.022).

When co-cross-linked with the γδ TCR the γδ T cell co-receptor WC1 becomes phosphorylated on key tyrosines and augments T cell responses (16). When those tyrosines are replaced through point mutations or WC1 is silenced the cells fail to respond (19, 20). As shown here, when wild-type WC1 was not silenced but rather separated from the TCR by using cross-linking with isotype-specific secondary Abs the cells also could not respond to stimulation through the TCR, while co-cross-linking them together using a general anti-IgG secondary Ab they were activated to proliferate (**Figure S2**). Thus, we hypothesized that WC1 and γδ TCR colocalize for cell activation to occur. To assess this, imaging flow cytometry (AMNIS) was used to measure FRET that occurs when molecules are ≤ 9 nm apart. As a pilot study to establish a baseline of FRET levels, we used two secondary Abs that react with different epitopes of an anti-WC1 mAb (**Figure S1A**). As a positive control using cells, we first evaluated CD3 and TCR interaction and found that after culture with *Leptospira* there was a visible shift in fluorescence indicating FRET had occurred (**Figure 1A**). In contrast, this did not occur with *ex vivo* quiescent cells. As a negative control CD45 and γδ TCR interaction was chosen for evaluation since those proteins are known to be in different protein islands both before and during activation of αβ T cells (28). Also, while CD45 is known to move into lipid rafts following activation of αβ T cells (29, 30) we found here that only about 20% of WC1 molecules in bovine γδ T cells were within lipid rafts and that none of the γδ TCR was (**Figure S2**). These latter results for WC1, γδ TCR and lipid rafts are in agreement with a previous study (18). As predicted, on δ TCR^+^ cells that constituted about ~8% of quiescent *ex vivo* cells and ~17% of cells from cultures stimulated with *Leptospira*, we found no FRET between CD45 and γδ TCR (**Figure 1B**). Finally for the principal experiment, when FRET between WC1 and γδ TCR on *ex vivo* resting, i.e., quiescent cells, was assessed none occurred (**Figure 1C**), while some lymphocytes cultured with *Leptospira* showed fluorescence sensitized emission of the acceptor fluorophore indicating FRET (**Figure 1C**). The cells that were positive for FRET had a clear shift in fluorescence and a demarcation was evident between stimulated and quiescent cells (**Figure 1D**). In contrast, with no primary antibody for the acceptor channel present, designed to measure the contribution of background autofluorescence, the two types of cells (*ex vivo* and cultured) had essentially complete overlap in fluorescence levels (**Figure S1B**). Statistical analysis of the 3 independent experiments showed significantly more FRET^+^ cells after *Leptospira* culture when CD3-TCR or WC1-TCR interactions were assessed but not between CD45 and TCR (**Figure 1E**). As an additional control, we found that secondary Ab alone showed few cells with background fluorescence that fell within the gates used to signify FRET^+^ cells (**Figure S1C**).

**Figure 2:**
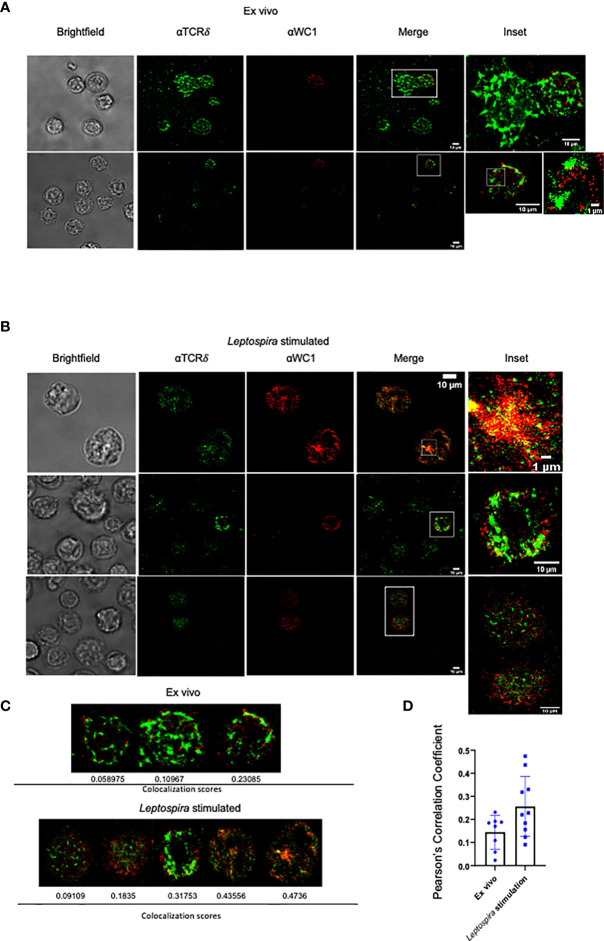
STORM imaging of bovine lymphocytes for WC1 and TCRδ interaction. Bovine PBMC were imaged by STORM after staining by immunofluorescence with anti-WC1 mAb (CC15) and AF647 isotype specific secondary Ab or anti-TCRδ mAb (GB21A) and AF488 isotype specific secondary Ab. Examples are shown here from 4 experiments conducted with two animals. **(A)** *Ex vivo* WC1^+^ γδ T cells and **(B)** WC1^+^ γδ T cells cultured with sonicated *Leptospira* antigen for 7 days. Cell size is indicated with white bars. The sub-cellular particle in Panel A is likely a RBC. **(C)** Examples of individual cells with their corresponding Pearson’s coefficient. **(D)** Comparison of Pearson’s coefficients from a larger sample of cells with the mean and SD shown.

We then examined the cells using STORM to obtain super-resolution fluorescence data on WC1 and γδ TCR interactions. On quiescent cells, there was a clear separation of WC1 and γδ TCR protein islands (**Figure 2A**). This was consistent with the literature regarding protein islands on resting lymphocytes in general (31). After culture with *Leptospira*, the WC1 and TCR protein islands became juxtaposed (**Figure 2B**) to various degrees (**Figure 2C**). Quantitative measurements of the colocalization were obtained through the corr2 MATLAB function to compute Pearson’s correlation coefficient. There was a significant difference between the *ex vivo* and *Leptospira* cultured groups (*p*=0.046). We found ~40% of the cells cultured with *Leptospira* had a significantly higher WC1 and TCR localization than quiescent *ex vivo* cells (**Figure 2D**). This agreed with the proportion of WC1^+^ γδ T cells from vaccinated animals that are known to undergo cell division following culture of PBMC with *Leptospira* as shown in previous studies (25).

### WC1 and γδ TCR Interactions on Cells in WC1 Subpopulations

**Figure 3:**
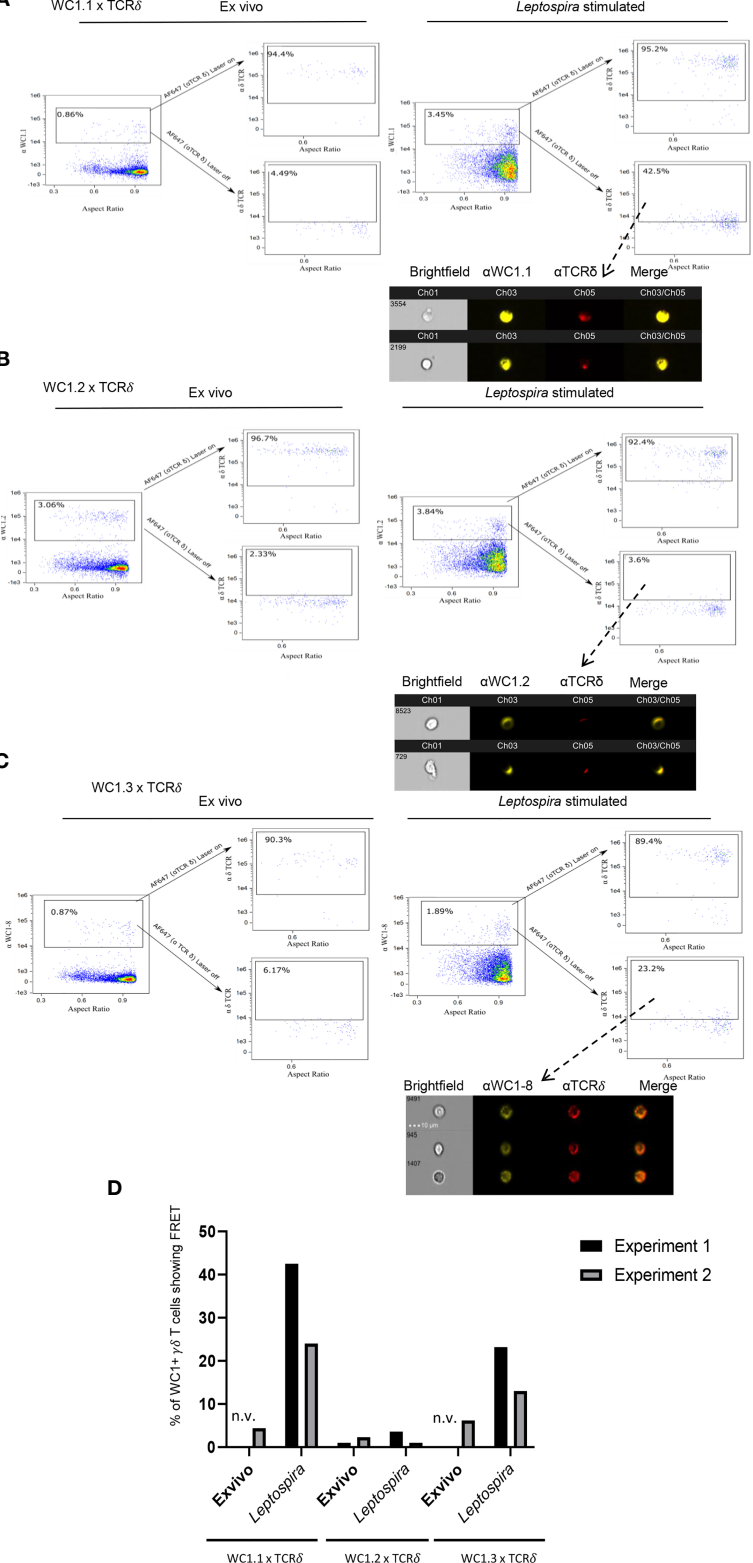
FRET between different variants of WC1 molecules and the γδ TCR. AMNIS imaging flow cytometry of bovine PBMC either *ex vivo* or following culture with *Leptospira* antigen for 7 days. For **(A–C)** the top flow cytometry plots to the right of the arrow indicate fluorescence with both lasers on, while the bottom panels to the right of the arrow show fluorescence with the red AF642-activating laser off. Individual cell pictures from the indicated (from dashed arrow) gated population are also shown. **(A)** Staining by indirect immunofluorescence with anti-WC1.1 mAb BAG25A with anti-IgM-PE Ab (donor) and anti-TCRδ mAb with anti-IgG2b-AF647 Ab (acceptor) or **(B)** anti-WC1.2 mAb CACTB32A with anti-IgG1-PE Ab (donor) and anti-TCRδ mAb with anti-IgG2b-AF647 Ab (acceptor). **(C)** Stained by indirect immunofluorescence with anti-WC1-8 (WC1.3) mAb CACT21A with anti-IgG1-PE Ab (donor) and anti-TCRδ mAb with anti-IgG2b-AF647 Ab (acceptor). AMNIS plots in panels **(A–C)** are representative of 2 experiments. **(D)** The % of cells showing FRET relative to the maximal number possible for the 2 experiments is shown in the bar graphs; n.v., not visible but was measured.

Unlike CD4 and CD8 coreceptors of αβ T cells, there are variants of WC1 molecules arising from the bovine WC1 multigenic array of 13 genes. There are multiple stable subpopulations of γδ T cells each expressing 1 to 6 different variants (32). These WC1 variants may differ in the number of extracellular SRCR domains as well as their endodomains (24). γδ T cells designated as WC1.1^+^ and WC1.2^+^ differ in the WC1 genes they express and in their responses to pathogens (22, 25, 32). For example, cells within the WC1.1^+^ γδ T cell subpopulation proliferate and produce IFN- γ in response to *Leptospira*, while many fewer WC1.2^+^ cells do (33). The WC1 variants designated as WC1.1-types (4) bind *Leptospira*, while the WC1.2-types do not (14), suggesting a reason for the difference in cellular response. Thus, we hypothesized that following stimulation with *Leptospira* the WC1 molecules on some cells within the WC1.1^+^ subpopulation would colocalize with the γδTCR, while WC1 molecules on fewer cells within the WC1.2^+^ population would do so. We found FRET occurred between the WC1 molecules that reacted with the anti-WC1.1 mAb BAG25A and the γδ TCR if cells had been stimulated with *Leptospira* in *in vitro* recall cultures (**Figure 3A**). In contrast, for WC1.2^+^ cells (identified by mAb CACTB32A) very few showed FRET between their WC1.2 molecules and the γδ TCR for either quiescent cells or those cultured with *Leptospira* (**Figure 3B**).

We next utilized a mAb against a single WC1 variant, WC1-8 (recognized by mAb CACT21A). WC1-8 is a leptospira-binding variant of WC1 expressed by cells within the WC1.1^+^ subpopulation. These specific cells are known as WC1.3^+^ and express the WC1-3 variant in addition to WC1-8. FRET occurred between WC1-8 and γδ TCR on cells cultured with *Leptospira* (**Figure 3C**) although it was less than occurred when the anti-WC1.1 mAb BAG25A (which reacts with WC1-3) or anti-pan-WC1 mAb CC15 (that is a pan-anti-WC1 mAb) was used. Interestingly, because mAb CACT21A was against a single WC1 variant (WC1-8), small microclusters of γδ TCR and WC1 were more distinct than WC1 clusters in experiments using the anti-pan-WC1 mAb CC15 (see **Figure 1C**). Quantitation showed that in both experiments performed there was consistent FRET between γδ TCR and WC1 on cells within the WC1.1^+^ and WC1.3^+^ subpopulations but not in the WC1.2^+^ subpopulation (**Figure 3D**).

### Different Variants of WC1 Molecules Interact Only After Cell Activation

**Figure 4:**
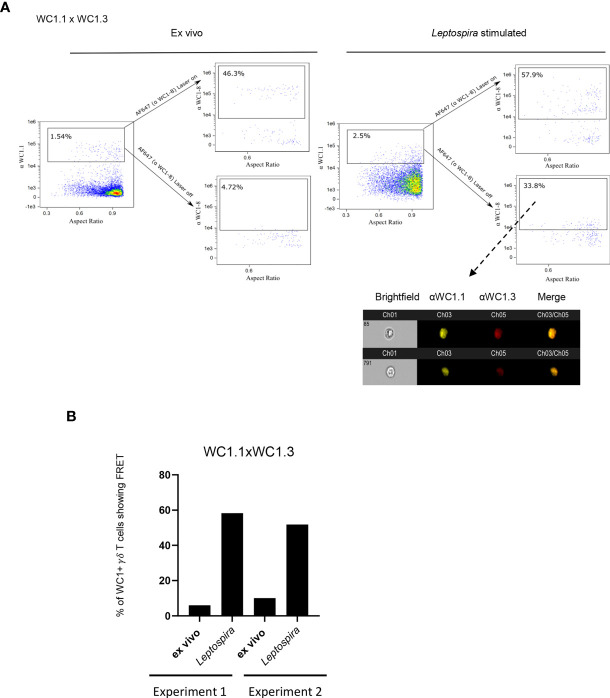
FRET between different variants of WC1 molecules. Bovine PBMC were either *ex vivo* or cultured with *Leptospira* antigen for 7 days and imaged with Amnis imaging flow cytometry. The top flow cytometry plots after the arrows indicate fluorescence with both lasers on as a positive control, while the bottom panels after the arrows show fluorescence with the red AF642- laser off to measure fluorescence-sensitized emission in that channel. Individual cell pictures from the indicated (dashed arrow) gated population are also shown. Cells were stained by immunofluorescence with **(A)** anti-WC1.1 mAb (BAG25A does not react with WC 1-8) with anti-IgM-PE Ab and anti-WC1-8 (i.e. WC1.3^+^ cells, mAb CACT21A) with anti-IgG1-AF647 secondary Ab. **(B)** The % of cells showing FRET relative to the maximal number possible is shown in the bar graphs for the 2 experiments performed.

We then evaluated whether the different WC1 variants act similarly to CD4 and cluster together despite their amino acid sequence and structural differences. To determine this, we evaluated FRET on a population of cells that are known to express just two specific variants of WC1 molecules. These cells are known as WC1.3^+^ γδ T cells, expressing WC1-8, recognized by mAb CACTB21A, and WC1-3, recognized by mAb BAG25A. There was no FRET between WC1 variants on the WC1.3^+^ cells when evaluated in their quiescent state (**Figure 4A**). Separation of these different WC1 variants on quiescent cells was affirmed with STORM using WC1.3^+^ flow cytometrically sorted cells (**Figure S4**). However, their pre-clustered protein islands containing either WC1-8 or WC1-3 become juxtaposed with one another after *Leptospira* stimulation (**Figure 4A**). FRET occurred between these WC1 variants in both of the experiments performed (**Figure 4B**).

### γδ TCR and CD3 Interact Prior to Interaction With WC1

**Figure 5:**
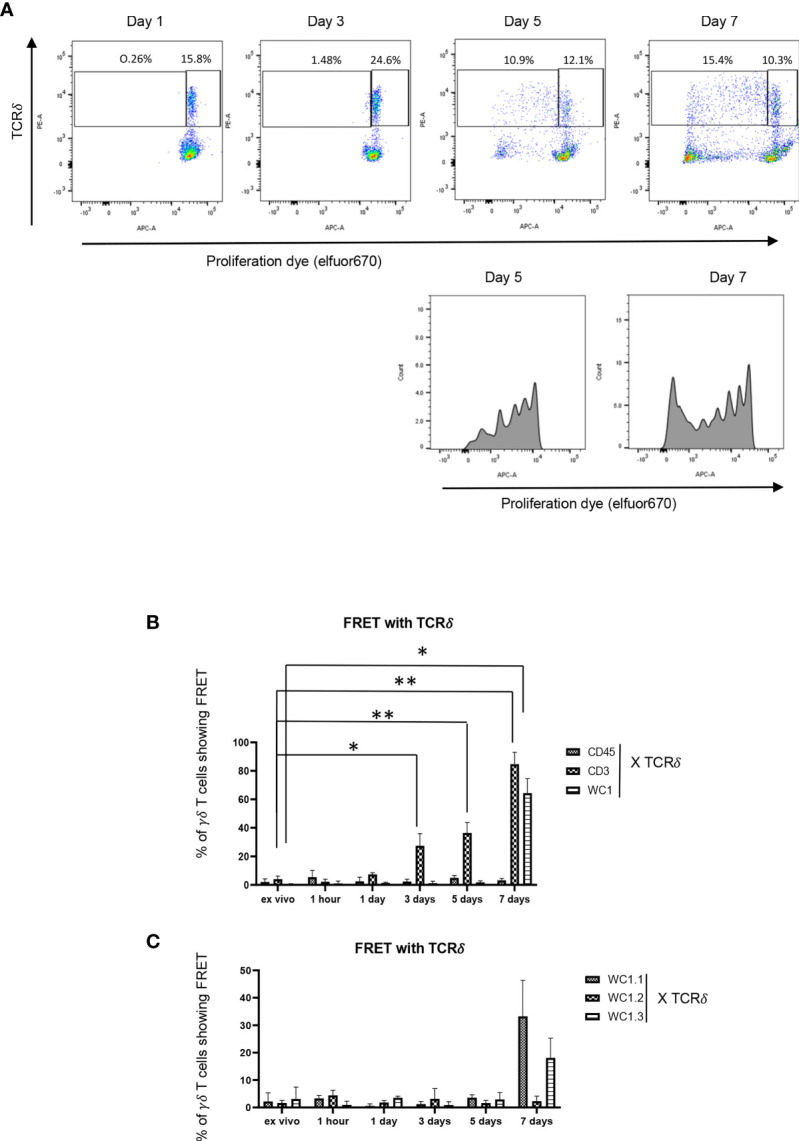
Temporal development of FRET between various cell surface molecules. **(A)** Flow cytometry of PBMC loaded with efluor670 cell division dye and then cultured with *Leptospira* sonicate for up to 7 days and stained by indirect immunofluorescence with anti-TCRδ mAb. **(B, C)** are results ofAMNIS imaging flow cytometry of bovine PBMC after culturing with *Leptospira* for variable lengths of time; indirect immunofluorescence with mAb as indicated included GB21A (anti-TCRδ), CC15 (anti-pan-WC1), CACTB32A (anti-WC1.2), BAG25A (anti-WC1.1), CACT21A (anti-WC1-8, marking WC1.3^+^ cells), and MM1A (anti-CD3) and appropriate isotype-specific secondary Abs. The mean percentage of γδ T cells showing FRET relative to the maximal number possible between the molecules is indicated in the bar graphs: **(B)** anti-CD45, anti-CD3 or anti-WC1 mAbs with anti-TCRδ mAb, **(C)** anti-WC1.1, anti-WC1.2 or anti-WC1.3 mAbs with anti-TCRδ mAb. **(B)** shows the mean ± SD of 3 independent experiments while **(C)** is the average of 2 experiments and thus no SD shown. Significant differences by Student’s t-test are shown (*p ≤ 0.05; **p ≤ 0.01) with specific values being: CD3 at three days *p* = 0.016, CD3 at five days *p* = 0.007, CD3 at seven days *p* = 0.005, and WC1 at seven days *p* = 0.022.

Cell division of γδ T cells stimulated with *Leptospira* antigen occurs by day 5 of culture (34) (**Figure 5A**). Using this as a guideline, we evaluated the temporal clustering of cell surface molecules following culture with *Leptospira*. There was no profound FRET above baseline observed between CD45 and γδ TCR throughout any of the timepoints (**Figure 5B**), while γδ T cells showed significant clustering of γδ TCR-CD3 complexes with FRET by day 3 (**Figure 5B**) and significant FRET between WC1 and the γδ TCR was measurable on day 7 when either a pan-anti-WC1 mAb (**Figure 5B**) or WC1 variant-specific mAbs (**Figure 5C**) were used. While we measured some cell division by day 5 of culture, additional cell proliferation had occurred by day 7 (**Figure 5A**) which agreed with the results showing WC1 and γδ TCR had increased FRET at day 7 (**Figures 5B, C**). Overall clustering of γδ TCR/CD3 complexes (as measurable by AMNIS) occurred earliest (day 3) before clustering of WC1 with the γδ TCR (day 7) in all the replicate independent experiments performed.

### *Leptospira* Binds to WC1 on γδ T Cells

**Figure 6:**
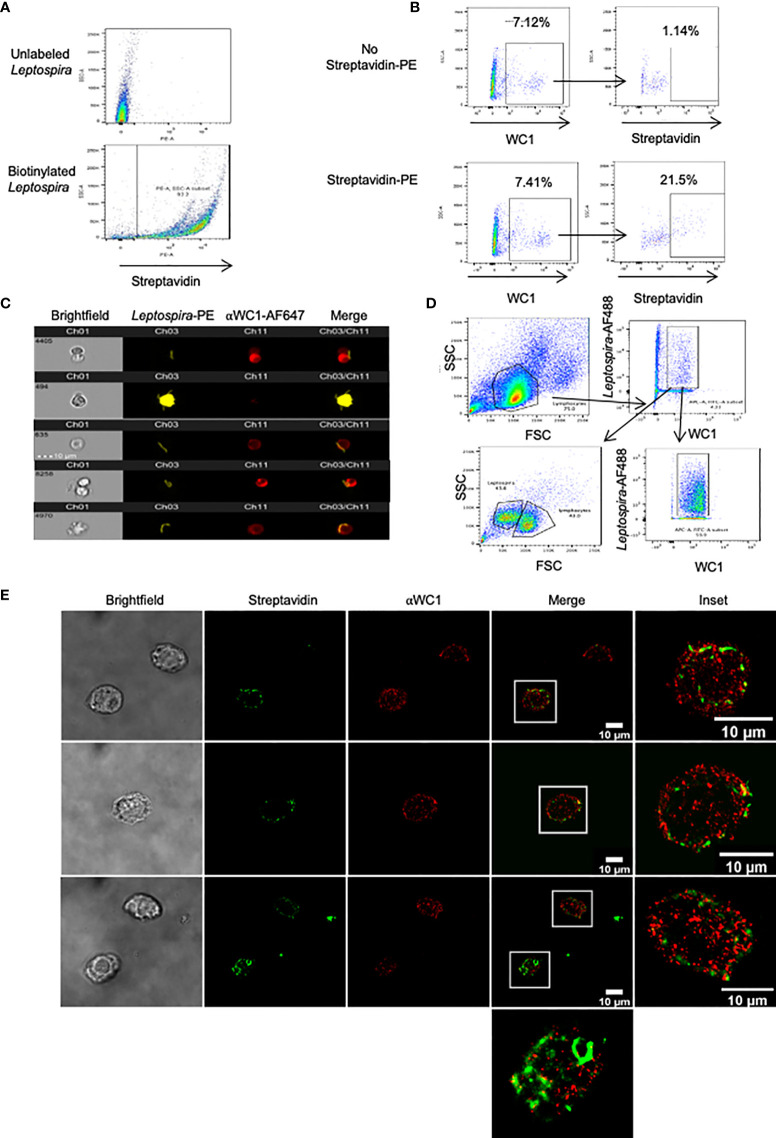
*Leptospira* binding to WC1^+^ cells. *Leptospira interrogans* serovar hardjo bacteria were biotinylated and stained with either streptavidin-PE or streptavidin-AF488. **(A)** Flow cytometry of unlabeled or biotinylated-streptavidin-PE *Leptospira* alone. **(B)** Bovine PBMC incubated with biotinylated *Leptospira* for 4 hr and stained by indirect immunofluorescence with anti-WC1 mAb CC15 and anti-IgG2a-AF647 secondary Ab. Top panels (unstained controls) had no streptavidin-PE added, while it was added to the bottom panels. **(C)** AMNIS images of double positive cells (WC1^+^/*Leptospira* ^+^) from **(B)**. **(D)** Flow cytometry sorting strategy of WC1^+^-AF647^+^ lymphocytes that bound biotinylated *Leptospira* ^+^-Steptavidin-AF488 Representative of 2 independent experiments performed. **(E)** Sorted cells shown in **(D)** were then imaged by STORM.

We next evaluated direct interaction of *Leptospira* with WC1 molecules using STORM. Since most studies have employed sonicated bacteria, we wanted to ensure γδ T cells cultured with whole *Leptospira* proliferated in recall responses; we found they did so with responses being even greater than with sonicated bacteria (**Figure S5A**). To determine whether leptospires bound to WC1 on γδ T cells we used biotinylated *Leptospira* (**Figure 6A**). When incubated with PBMC we found that the WC1^+^ cells bound *Leptospira* (**Figure 6B**) with one or more leptospires as shown by AMNIS imaging flow cytometry (**Figure 6C**). To determine whether WC1 and *Leptospira* were juxtaposed on the cell surface STORM imaging of flow cytometrically sorted WC1^+^ cells with bound bacteria was done (**Figure 6D**). There was some colocalization between WC1 and the bacteria evident as indicated by yellow overlay (**Figure 6E**). The bacteria were more difficult to image as shown by their discontinuous appearance (see **Figure S5B** *, AMNIS images of bacteria*) due to their size which was considerably larger than the protein islands in quiescent cells.

**Figure 7:**
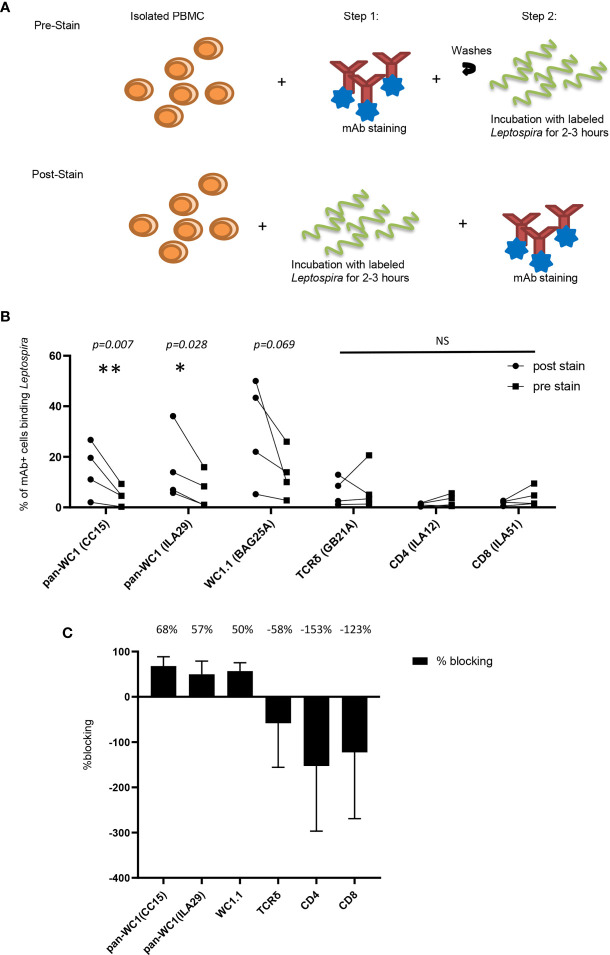
Blocking of Leptospira binding to lymphocytes by anti-WC1 Ab. **(A)** *Leptospira interrogans* serovar hardjo was biotinylated and stained with Streptavidin-PE. Experimental design of blocking adherence of bacteria to cells by mAbs added either before (pre-stained) or after (post-stained) incubation with the bacteria. **(B)** Cells post-stained with the indicated mAbs reactive with lymphocyte surface antigens and isotype-specific secondary Abs were compared to those pre-stained before the 3-hr incubation with *Leptospira*-biotin-streptavidin-PE. Percentages are from gated populations of mAb^+^ cells and evaluated for *Leptospira* binding from that population. The results show the binding of bacteria for the indicated lymphocyte population. Lines connect the results within an experiment (n=4 independent experiments). Significant differences were done by the Mann-Whitney ranked sum (*p ≤ 0.05 **p ≤ 0.01; mAb CC15 *p* = 0.007, mAb BAG25A *p* = 0.069 and mAb ILA29 *p* = 0.028) NS, not significant. **(C)** Percentage of blocking by the mAbs in **(B)** was the difference between percentage of cells binding *Leptospira* post-stained and pre-stained. A positive number indicates blocking, while a negative number indicates enhanced binding. The mean percentage of blocking is indicated above each treatment.

*Leptospira* is known to bind to a variety of cells as well as to extracellular matrix through adhesins (35). To ensure that binding to γδ T cells was due to the interaction with WC1 the bacteria were incubated with lymphocytes before and after the lymphocytes were coated with mAb to block cell surface molecules including several anti-WC1 mAbs (**Figure 7A**). We found that mAb against WC1, when used before incubating the cells with the *Leptospira*, blocked binding of the bacteria to the cell (**Figure 7B**). This varied from experiment to experiment (4 independent experiments performed) due to variation in the level of bacterial binding. However, while blocking with anti-WC1 mAbs was consistent in all 12 evaluations no consistent blocking by mAbs that bind to TCRδ, CD4, or CD8 occurred (**Figure 7C**). The anti-pan-WC1 mAb CC15 had the best blocking ability, blocking nearly 70% of *Leptospira* binding and the blocking was statistically significant.

## Discussion

We hypothesized that since WC1 and γδ TCR augment signaling when co-ligated together (15) and both are needed for γδ T cell activation (16, 22, 36, 37) that they would cluster together in the cell membrane following activation with *Leptospira*, the model used in our studies (20, 23, 34, 38). Using AMNIS imaging flow cytometry and then STORM for higher resolution imaging we showed that the WC1 co-receptors colocalized with the γδ TCR on activated cells resulting in FRET which indicates they were within 9 nm of one another. WC1, like the TCR co-receptor CD4 as well as many other cell surface molecules, was found in protein islands or nano or microclusters on the cell membranes of quiescent γδ T cells and those islands then concatamerized with protein islands containing γδ TCR following activation of the cells. Individual γδ T cells may express more than one WC1 gene from the multigenic array (32); here we showed that homologous types of WC1 molecules clustered together in resting cells but then concatenated with islands containing other WC1 variants following cell activation. This occurred before the coalesced heterogeneous clusters of WC1 variants merged with the γδ TCR protein islands. We also found that *Leptospira* spirochetes bound specifically to WC1 rapidly on the surface of γδ T cells when cultured together. These observations support the concept of a signaling domain forming which contains WC1 with the TCR along with the ligand. This could indicate that both the WC1 and TCR bind the ligand or an interaction between WC1 and TCR similar to what has been shown for butyrophilin’s interaction with the germline-encoded portion of the human Vγ1 chain (39). It is possible that this complex is later endocytosed together to limit cell activation since we know from longer term studies of T central memory cells derived from cultures similar to those used here have a decreased MFI of the γδTCR and WC1 (Gillespie, unpublished data).

Unexpectedly, we found CD3 and γδ TCR were not close enough for FRET in quiescent cells although they were following culture of the cells with *Leptospira*. We postulate that following cell activation FRET may have been caused by a trans mechanism when other complexes of TCR-CD3 form tighter clusters together. This is consistent with αβ T cell immune synapse formation in that TCR-CD3 complexes are found to more tightly associate after initial stimulus (40). Also, because of the position of the antibody epitopes on CD3 and TCR, combined with the use of indirect immunofluorescence, the distances between these molecules may have been extended further than if directly conjugated antibodies had been used. There are also fundamental differences in the CD3 of αβ and γδ T cells that could contribute to this result. For example, we have previously shown that plate-bound anti-CD3 antibodies cause most bovine αβ T cells to become activated and proliferate but very few γδ T cells do (41). This has been confirmed by others (42). Also, most quiescent γδ T cells in mice, unlike αβ T cells, do not express CD3δ (43) but instead express a glycosylated form of CD3γ following activation (44). In addition, when αβ T cells are stimulated they have a conformational change associated with their CD3ϵ that is required for activation (45) but γδ T cells do not do this (46). Finally, the γδ TCR is oriented differently to the cell membrane than the αβ TCR is (47) consistent with the difference in the types of antigens that γδ T cells recognize, which are not peptides presented on MHC.

Group B SRCR superfamily members include CD5, CD6 and CD163 that are known to bind bacteria and fungi through their extracellular SRCR domains (11–13). In cattle, there are 13 WC1 molecules with 6 or 11 SRCR extracellular domains, each of which can potentially bind pathogens (14). We have shown that multiple serovars of *L. interrogans* as well as *L. borgpetersenii* can bind to specific recombinant WC1 SRCR domains in solution and that this binding is sensitive to specific amino acid mutation (14) and that 75-80% of *Leptospira*-responding WC1^+^ γδ T cell clones have transcripts for at least one WC1 molecule that has the potential to bind *Leptospira* (32). Here we showed direct binding to the WC1 proteins on γδ T cell membranes. It is known that *Leptospira* spp. can bind epithelial cells as well as the extracellular matrix (48). Indeed, we found here that *Leptospira* could bind to some cells in PBMC nonspecifically. However, in the case of WC1^+^ γδ T cells the bacteria binding was specific for WC1 as shown by anti-WC1 mAb blocking but not for example blocking by the anti-TCRδ mAb supporting our previous work.

While individual γδ T cells express up to 6 variants of WC1 (32), it was unclear whether all WC1 variants on the cell would colocalize together regardless of whether they bound bacteria or not. We were able to increase our understanding of this showing that while in quiescent cells the variants of WC1 are in separate and spatially stable protein domains or islands that following activation the island with different variants coalesce and then merge with the γδ TCR islands. This occurred coincident with the time of the first cell division. This suggests that following cell activation that the separate WC1 protein islands cluster more tightly before concatermization with the γδ TCR islands. Because WC1 has variegated gene expression (24, 32), the WC1.2^+^ cells that divided could occasionally be expressing WC1.1 variants subpopulation that bind leptospires, as we have shown using γδ T cell clones (32), and thus why they were found among the dividing cells. Despite differences among individual WC1 molecules they all have the capacity for signal transduction and thus based on these data we hypothesize that they contribute to cell activation once clustered together.

With regard to signaling and cell activation, we have shown in the past that WC1 has src family tyrosine phosphorylation sites and that key tyrosines in their endodomains are phosphorylated within 30 seconds following co-crosslinking of WC1 with the TCR (16, 19). Kinases able to phosphorylate WC1 included fyn, blk, and lyn (16, 49); these may associate with WC1 to play this role in activation following clustering of WC1 and the γδ TCR. We also expect molecules associated with an immune synapse of an αβ T cells such as lck or zap70 (50) as well as src kinases to similarly associate with the molecular clustering of γδ TCR and WC1. Because γδ T cells do not react with antigenic peptides associated with MHC on antigen presenting cells construction of a SMAC may not necessarily be possible. It was previously believed that T cells need a cSMAC for TCR-mediated activation but more recently shown this is not necessarily the case since this is not required of naive CD8 T cells (51). Also, others contend that the SMAC does not have a role in long term TCR activation but instead plays a role in down-regulation of signal (52). While a classical immune synapse may not form on γδ T cells, nevertheless, here we found that the clustering of receptors in the membranes on the γδ T cells shared some of the core attributes in that CD45 was excluded from the protein islands of the TCR. The temporal relationship of the events described here is more perplexing. The proximity and time required for FRET to occur suggests that WC1 and γδ TCR physically interact in ways that differ from that seen by the αβ TCR and the TCR co-receptors CD4 and CD8. However, it is particularly important to note that here we used *ex vivo* PBMC, while other studies used pre-cultured cells or cell lines (31, 53) including our own studies of phosphorylation events which used WC1-transfected Jurkat cells that showed events within seconds (16). When T cells from a transgenic mouse where evaluated, TCR protein island clustering could not be seen until 2.5 - 5 hours (54) although this is still considerably earlier than our observations. Since we saw no division until day five of culture the activation events measured in other studies of αβ T cells would be expected to be quite different from those in the heterogenous population of γδ T cells used here. An understanding of how these WC1^+^ γδ T cells signal and respond to pathogens may potentiate development of vaccines that recruit and stimulate these cells by exploiting the role of the WC1 co-receptors. While open questions remain, the studies performed here may shed light on that.

## Data Availability Statement

The original contributions presented in the study are included in the article/**Supplementary Material**. Further inquiries can be directed to the corresponding author.

## Ethics Statement

The animal study was reviewed and approved by University of Massachusetts Amherst Institutional Animal Care and Use Committee.

## Author Contributions

AG, MG, and TS conducted the experiments, analyzed the data and contributed to writing of the manuscript. TC, JT, and CB conceived of the experimental approach, secured the funding and contributed to analyzing data and writing the manuscript. All authors contributed to the article and approved the submitted version.

## Funding

This project was supported by AFRI Grant #2016-09379 from the USDA-National Institute of Food and Agriculture to CB, JT, and TC, Hatch funding from the Umass Center for Agriculture, Food and the Environment under project #2016-67015-24913 to CB and JT, NIH grant #HD-038082 to P. Visconti for funding MG and Hong Family Fellowship to AG. The contents are solely the responsibility of the authors and do not necessarily represent the official views of the USDA, NIFA or NIH.

## Conflict of Interest

The authors declare that the research was conducted in the absence of any commercial or financial relationships that could be construed as a potential conflict of interest.

## Publisher’s Note

All claims expressed in this article are solely those of the authors and do not necessarily represent those of their affiliated organizations, or those of the publisher, the editors and the reviewers. Any product that may be evaluated in this article, or claim that may be made by its manufacturer, is not guaranteed or endorsed by the publisher.

## Supplementary Material

Supplementary Figure 1 Controls for FRET. **(A)** PBMC were stained by indirect immunofluorescence with an anti-WC1 mAbs and secondary antibodies (αIgG-PE and αIgG2a-AF647) that react with 2 different epitopes on the mouse mAb and analyzed by AMNIS imaging flow cytometry as a pilot study to establish FRET measurement. **(B)** To evaluate autofluorescence in the AF647 channel, either *ex vivo* PBMC or from 7-day cultures with *Leptospira*, were stained with the primary anti-WC1 mAb CC15 (IgG2a) and anti-IgG2a-PE and anti-IgG2b -AF647 secondary Abs and assessed with the AF647 laser off. **(C)** AMNIS imaging flow cytometry of *ex vivo* PBMC stained with the indicated secondary Ab alone (anti-IgG-PE, anti-IgG2b-AF647, or anti-IgG2a-PE). Gates shown are those in which FRET^+^ cells occurred. Pictures of the few cells that were found in the gates that indicated FRET are indicated by arrow and images shown.

Supplementary Figure 2 WC1 and TCR crosslinking. Bovine PBMC cultured for 4 days with antibody combinations indicated below the x-axis and evaluated for ^3^H-thymidine incorporation. Each condition was set-up in triplicate wells. The results are expressed as the mean ± SEM of 3 cultures. Isotype-specific secondary antibodies resulted in capping of the individual molecule types, while the anti-IgG resulted in co-crosslinking of TCR and WC1 together. Statistically significant differences were evaluated by Student’s t-test and shown on the figure.

Supplementary Figure 3 Association of cell surface molecules with lipid rafts. AMNIS imaging flow cytometry of bovine PBMC either *ex vivo* or stimulated with *Leptospira* sonicate for 7 days. Cells were stained by mAb CC15 with IgG2a-AF647, or mAb GB21A with IgG2b-AF647 and lipid raft marker Cholera toxin B. Flow cytometry gating on *ex vivo* cells is shown and similar gating was done for *Leptospira* cultured cells (not shown). Representative of 2 experiments.

Supplementary Figure 4 STORM of resting WC1.1+/WC1.3+ cells *Ex vivo* PBMC were stained by anti-WC1.1 mAb (BAG25A) with anti-IgM-AF647 secondary Ab and by anti-WC1-8 (i.e., anti-WC1.3, mAb CACT21A) with anti-IgG1-AF488 secondary Ab. Cells were then sorted by flow cytometry for double positive cells and imaged with STORM.

Supplementary Figure 5 γδ T cells stimulation by intact bacteria. **(A)** *Ex vivo* PBMC were loaded with efluor670 dye to track cell divisions and then cultured for 7 days with either 0.08 mg/ml sonicated *Leptospira* or 5 x 10^5^/ml fixed intact *Leptospira* bacteria. PBMC were stained by indirect immunofluorescence by anti-TCRδ mAb with anti-IgG2b-PE secondary Ab and analyzed by flow cytometry. Percentage of dividing TCRδ^+^ cells are shown in the boxes. **(B)** *Leptospira interrogans* serovar Hardjo bacteria were biotinylated and stained with streptavidin-AF488 and imaged with STORM.

## Abbreviations

Ab, antibody; AF, alexafluor; ConA, Concanavalin A; FBS, fetal bovine serum; FRET, fluorescence resonance energy transfer; IFN, interferon; mAb, monoclonal antibody; PBMC, peripheral blood mononuclear cells; PE, phycoerythrin; PRR, pattern recognition receptor; cRPMI, complete RPMI; SRCR, scavenger receptor cysteine rich; cSMAC, central supramolecular activation complex; STORM, stochastic optical reconstruction microscopy; TCR, T cell receptor; WC1, workshop cluster 1.
