# Supplementary figures and images for: Gamma Delta TCR and the WC1 Co-Receptor Interactions in Response to Leptospira Using Imaging Flow Cytometry and STORM
Source: Front Immunol. 2021 Jul 28;12:712123. doi: 10.3389/fimmu.2021.712123 (PMC8356672; doi:10.3389/fimmu.2021.712123)

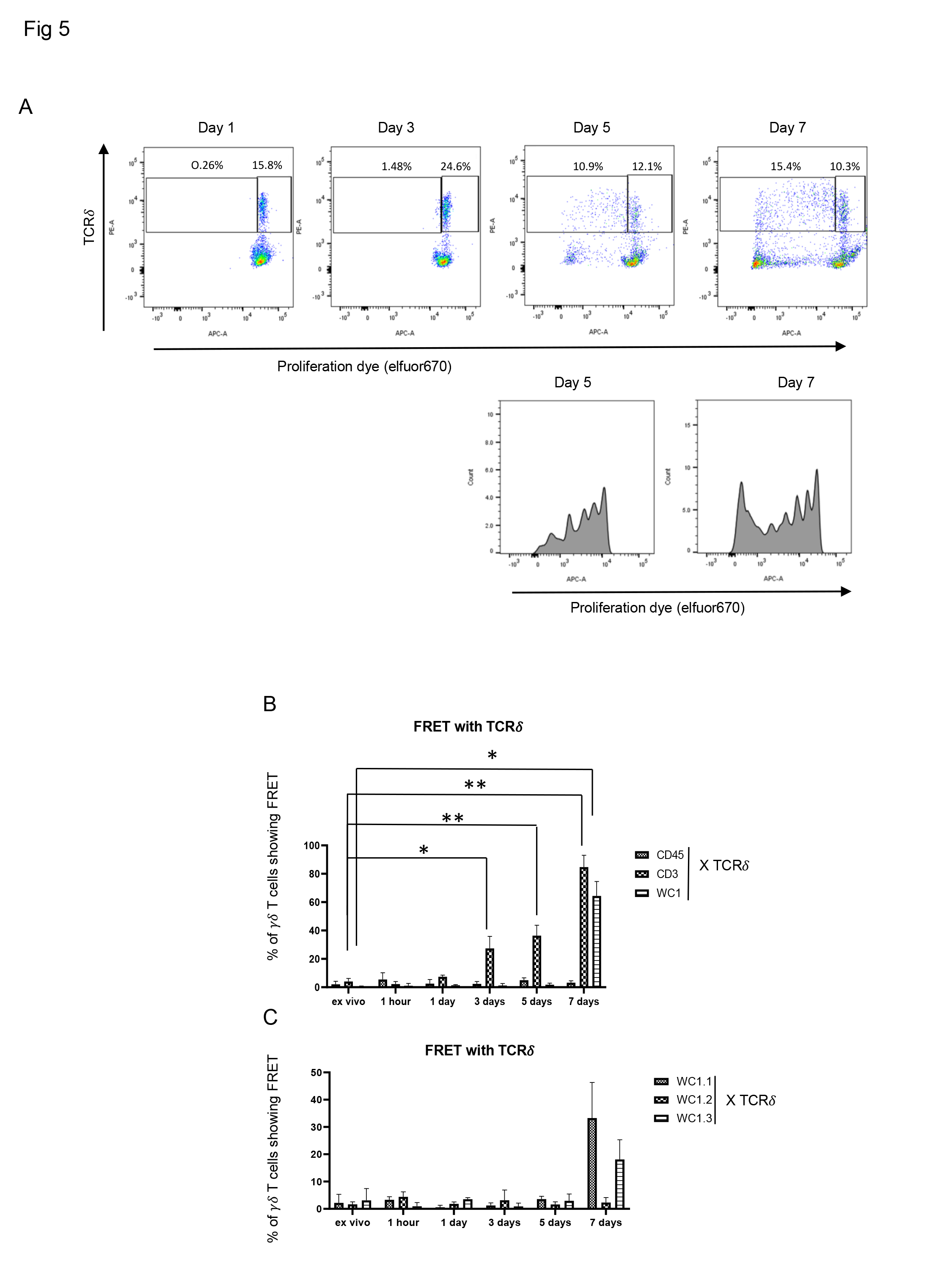

Supplement: Supplementary Figure 1 — Controls for FRET. (A) PBMC were stained by indirect immunofluorescence with an anti-WC1 mAbs and secondary antibodies (αIgG-PE and αIgG2a-AF647) that react with 2 different epitopes on the mouse mAb and analyzed by AMNIS imaging flow cytometry as a pilot study to establish FRET measurement. (B) To evaluate autofluorescence in the AF647 channel, either ex vivo PBMC or from 7-day cultures with Leptospira, were stained with the primary anti-WC1 mAb CC15 (IgG2a) and anti-IgG2a-PE and anti-IgG2b -AF647 secondary Abs and assessed with the AF647 laser off. (C) AMNIS imaging flow cytometry of ex vivo PBMC stained with the indicated secondary Ab alone (anti-IgG-PE, anti-IgG2b-AF647, or anti-IgG2a-PE). Gates shown are those in which FRET+ cells occurred. Pictures of the few cells that were found in the gates that indicated FRET are indicated by arrow and images shown. [file Image_1.tiff]

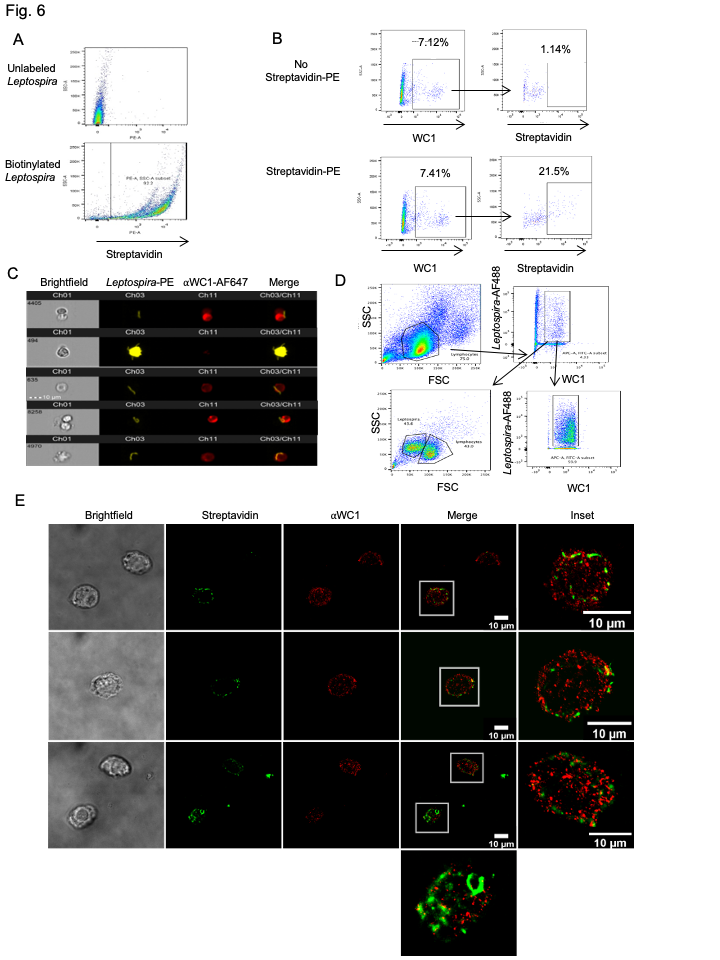

Supplement: Supplementary Figure 2 — WC1 and TCR crosslinking. Bovine PBMC cultured for 4 days with antibody combinations indicated below the x-axis and evaluated for 3H-thymidine incorporation. Each condition was set-up in triplicate wells. The results are expressed as the mean ± SEM of 3 cultures. Isotype-specific secondary antibodies resulted in capping of the individual molecule types, while the anti-IgG resulted in co-crosslinking of TCR and WC1 together. Statistically significant differences were evaluated by Student’s t-test and shown on the figure. [file Image_2.tiff]

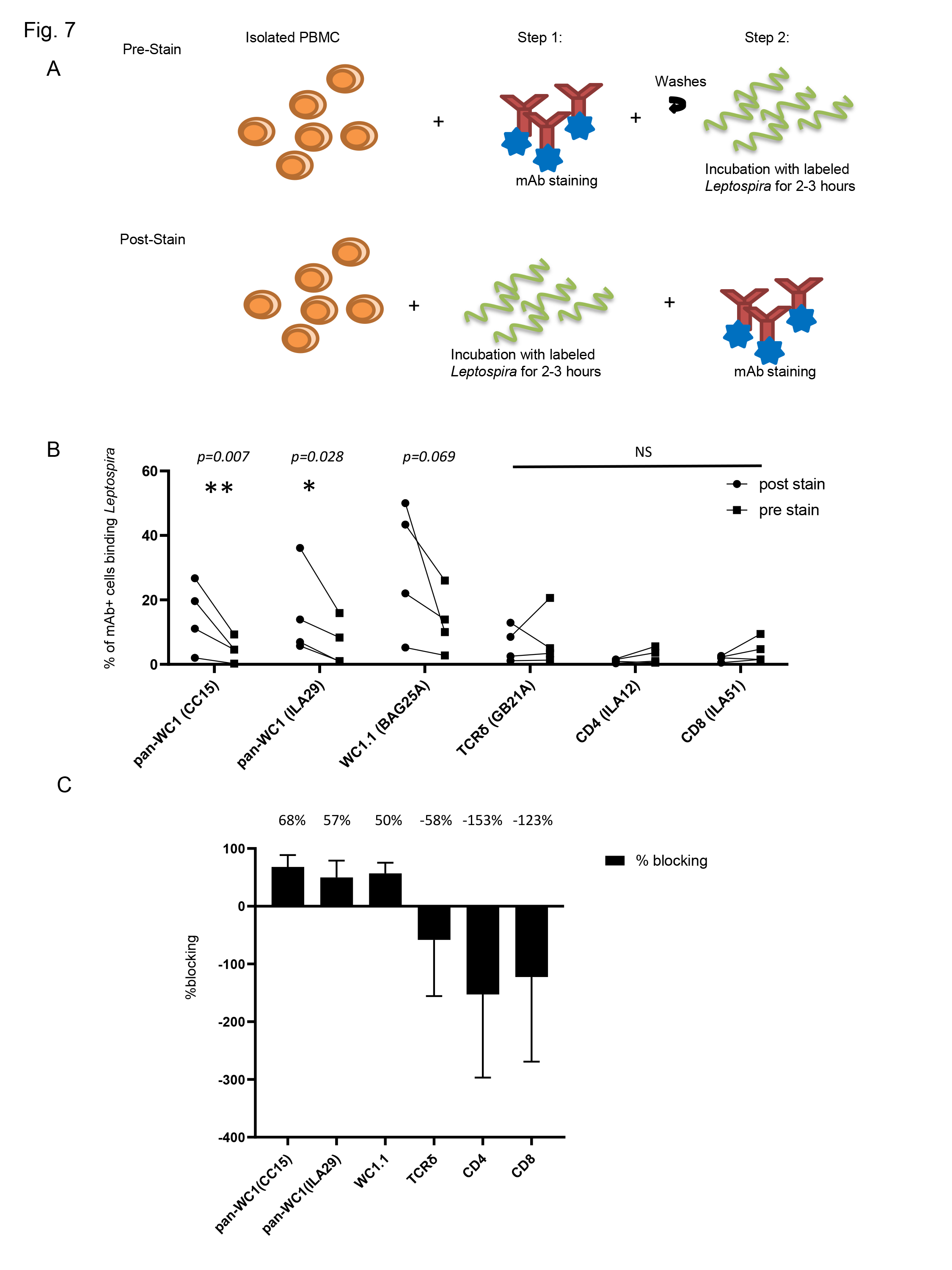

Supplement: Supplementary Figure 3 — Association of cell surface molecules with lipid rafts. AMNIS imaging flow cytometry of bovine PBMC either ex vivo or stimulated with Leptospira sonicate for 7 days. Cells were stained by mAb CC15 with IgG2a-AF647, or mAb GB21A with IgG2b-AF647 and lipid raft marker Cholera toxin B. Flow cytometry gating on ex vivo cells is shown and similar gating was done for Leptospira cultured cells (not shown). Representative of 2 experiments. [file Image_3.tiff]

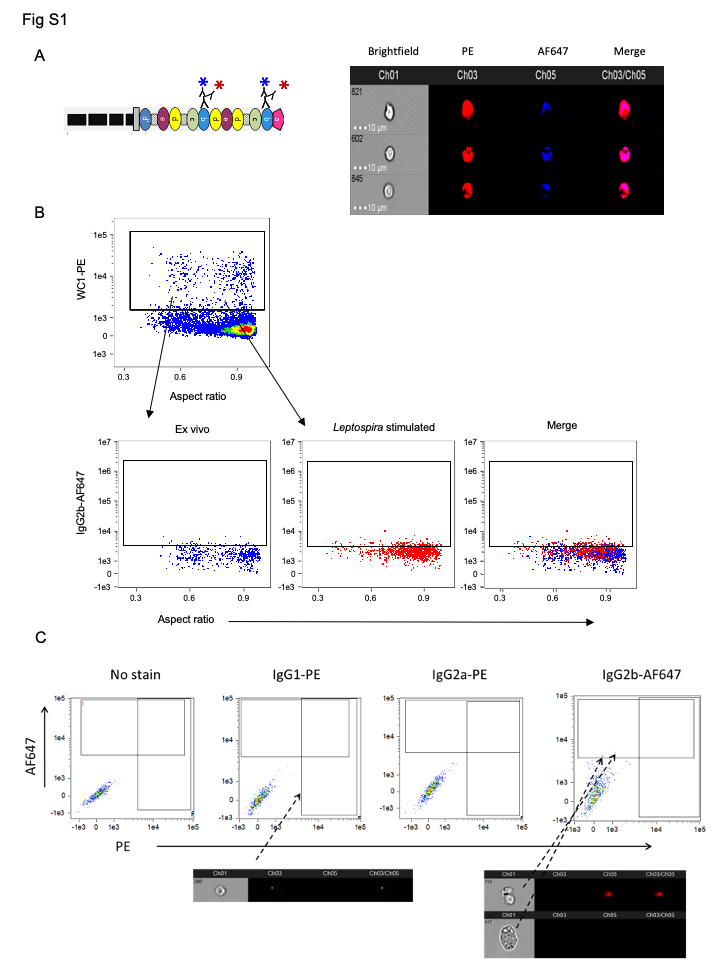

Supplement: Supplementary Figure 4 — STORM of resting WC1.1+/WC1.3+ cells Ex vivo PBMC were stained by anti-WC1.1 mAb (BAG25A) with anti-IgM-AF647 secondary Ab and by anti-WC1-8 (i.e., anti-WC1.3, mAb CACT21A) with anti-IgG1-AF488 secondary Ab. Cells were then sorted by flow cytometry for double positive cells and imaged with STORM. [file Image_4.tiff]

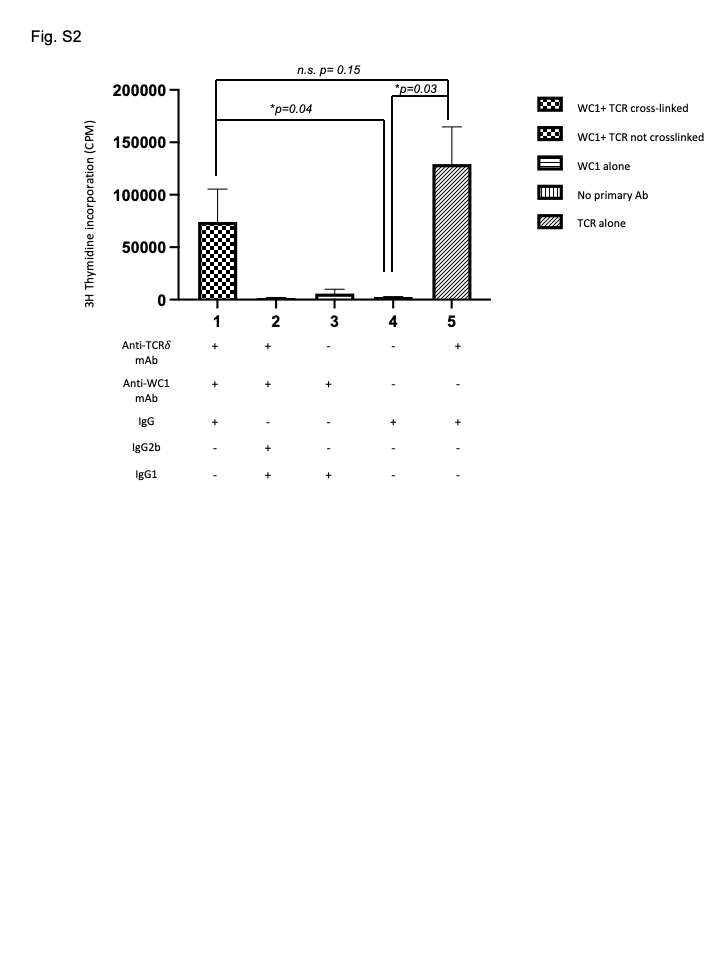

Supplement: Supplementary Figure 5 — γδ T cells stimulation by intact bacteria. (A) Ex vivo PBMC were loaded with efluor670 dye to track cell divisions and then cultured for 7 days with either 0.08 mg/ml sonicated Leptospira or 5 x 10^5^/ml fixed intact Leptospira bacteria. PBMC were stained by indirect immunofluorescence by anti-TCRδ mAb with anti-IgG2b-PE secondary Ab and analyzed by flow cytometry. Percentage of dividing TCRδ+ cells are shown in the boxes. (B) Leptospira interrogans serovar Hardjo bacteria were biotinylated and stained with streptavidin-AF488 and imaged with STORM. [file Image_5.tiff]
